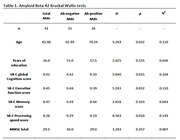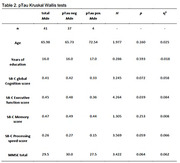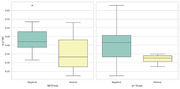# Identification of underlying AD pathology in cognitively unimpaired individuals with subjective cognitive decline by the means of automated speech and language processing

**DOI:** 10.1002/alz.094373

**Published:** 2025-01-09

**Authors:** Gonzalo Sánchez‐Benavides, Felix Doerr, Andreea Rădoi, Oriol Grau‐Rivera, Carolina Minguillon, Carlota Medina Rodríguez, Karine Fauria, Johannes Tröger, Nicklas Linz, Juan Domingo Gispert, Alexandra König

**Affiliations:** ^1^ Barcelonaβeta Brain Research Center (BBRC), Barcelona Spain; ^2^ ki:elements GmbH, Saarbrücken Germany; ^3^ Barcelonaβeta Brain Research Center (BBRC), Pasqual Maragall Foundation, Barcelona Spain; ^4^ ki elements UG, Saarbrücken Germany

## Abstract

**Background:**

Changes in speech and language functions have shown to be early symptoms of AD pathology. Recent developments in automatic speech and language processing have opened avenues for objective assessments of these changes. The primary objective of this study is to explore whether speech and language markers extracted from cognitive testing conducted during an automated phone call differ according to underlying AD pathology as measured in cerebrospinal fluid (CSF) in preclinical or early stage individuals.

**Method:**

Within the PROSPECT‐AD project, speech and gold‐standard clinical and biomarker data were obtained from the Beta‐AARC cohort in Barcelona, Spain. The sample consisted of N = 41 cognitively unimpaired Spanish and Catalan speakers with Subjective Cognitive Decline. Speech features were extracted from phone recordings of the Rey Auditory Verbal Learning Task (RAVLT) and the Semantic Verbal Fluency Task (SVF). The Speech‐Biomarker for Cognition (SB‐C) was computed based on 43 temporal and semantic features. Based on established cutoffs, the sample was divided into amyloid beta 42 positive and negative and pTau positive and negative groups. Kruskal‐Wallis tests were used to compare the positive and negative groups with regards to their overall cognition score and the SB‐C subscores memory, executive function and processing speed for each of the CSF biomarkers respectively.

**Results:**

The results of the group comparisons with regards to the amyloid beta 42 and pTau groups can be found in Table 1 and Table 2 respectively. Corresponding boxplots are presented in Figure 1. In summary, the amyloid beta negative group scored significantly higher on the SB‐C than the amyloid beta positive group. In particular, the difference had a strong effect size for the processing speed subscore and a medium effect size for the executive function subscore. No significant difference was found with regards to the memory subscore. For the pTau groups, the difference in overall cognition score showed a trend towards significance.

**Conclusion:**

This approach could establish a non‐invasive and accessible means of identifying potential indicators of AD, contributing to early detection and timely intervention strategies.